# Mass Spectrometry Based Identification of Geometric Isomers during Metabolic Stability Study of a New Cytotoxic Sulfonamide Derivatives Supported by Quantitative Structure-Retention Relationships

**DOI:** 10.1371/journal.pone.0098096

**Published:** 2014-06-03

**Authors:** Mariusz Belka, Weronika Hewelt-Belka, Jarosław Sławiński, Tomasz Bączek

**Affiliations:** 1 Department of Pharmaceutical Chemistry, Faculty of Pharmacy, Medical University of Gdańsk, Gdańsk, Poland; 2 Department of Analytical Chemistry, Chemical Faculty, Gdańsk University of Technology, Gdańsk, Poland; 3 Mass Spectrometry and Chromatography Laboratory, Pomeranian Science and Technology Park, Gdynia, Poland; 4 Department of Organic Chemistry, Faculty of Pharmacy, Medical University of Gdańsk, Gdańsk, Poland; Swiss Institute of Bioinformatics, Switzerland

## Abstract

A set of 15 new sulphonamide derivatives, presenting antitumor activity have been subjected to a metabolic stability study. The results showed that besides products of biotransformation, some additional peaks occurred in chromatograms. Tandem mass spectrometry revealed the same mass and fragmentation pathway, suggesting that geometric isomerization occurred. Thus, to support this hypothesis, quantitative structure-retention relationships were applied. Human liver microsomes were used as an *in vitro* model of metabolism. The biotransformation reactions were tracked by liquid chromatography assay and additionally, fragmentation mass spectra were recorded. *In silico* molecular modeling at a semi-empirical level was conducted as a starting point for molecular descriptor calculations. A quantitative structure-retention relationship model was built applying multiple linear regression based on selected three-dimensional descriptors. The studied compounds revealed high metabolic stability, with a tendency to form hydroxylated biotransformation products. However, significant chemical instability in conditions simulating human body fluids was noticed. According to literature and MS data geometrical isomerization was suggested. The developed *in sillico* model was able to describe the relationship between the geometry of isomer pairs and their chromatographic retention properties, thus it supported the hypothesis that the observed pairs of peaks are most likely geometric isomers. However, extensive structural investigations are needed to fully identify isomers’ geometry. An effort to describe MS fragmentation pathways of novel chemical structures is often not enough to propose structures of potent metabolites and products of other chemical reactions that can be observed in compound solutions at early drug discovery studies. The results indicate that the relatively non-expensive and not time- and labor-consuming *in sillico* approach could be a good supportive tool assisting the identification of *cis-trans* isomers based on retention data. This methodology can be helpful during the structural identification of biotransformation and degradation products of new chemical entities - potential new drugs.

## Introduction

Sulphonamides play an important role in medicine as antimicrobials (*e.g*., sulfamethoxazole), thiazide diuretics (*e.g*., indapamide), loop diuretics (*e.g*., furosemide), anti-diabetic sulfonylureas (*e.g.*, glipizide), some COX-2 inhibitors (*e.g*., celecoxib), and antiglaucoma agents such as acetazolamide.

**Figure 1:**
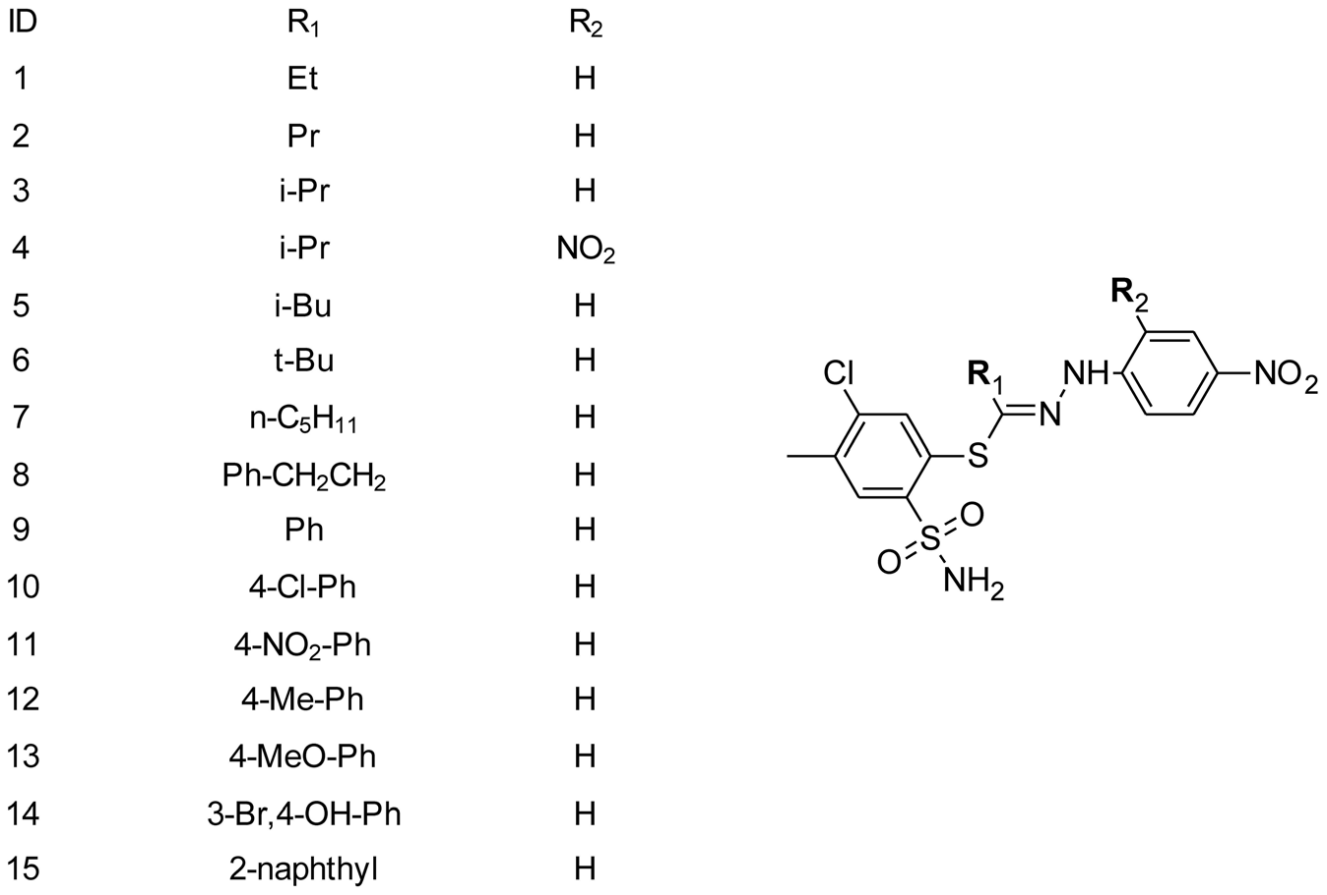
Chemical structures of the 15 studied compounds.

There is a growing interest, in the field of medicinal chemistry, in sulfonamides as potential anticancer agents [1], [2]. Carbonic anhydrases (CA), pointed out as potential sulphonamide targets, are enzymes catalyzing the interconversion between carbon dioxide and bicarbonate with the generation of protons. The carbonic anhydrase isoenzyme IX is highly overexpressed in hypoxic tumors and presents limited expression in normal tissues, so it is indicated as a molecular target, which can be inhibited by some sulfonamides [3]. Pazopanib [4] and indisulam [5], [6] are examples of sulfonamides in the clinical development of cancer treatment. Recently, a few series of biologically active sulphonamides were synthesized at the Department of Organic Chemistry at the Medical University of Gdańsk [7]–[11]. The structures of compounds studied in this paper are presented in Fig. 1. The set of 15 compounds belongs to a sulphonamide group, but additionally possesses thiohydrazone moiety, unique for its known anticancer compounds. Their synthesis, activity and their relationship between cytotoxic activity and structure were described in our previous reports [8], [12], [13].

Within the ADME processes, metabolism certainly covers the largest, and still poorly understood, aspect and consequently the most difficult to evaluate and to predict [14]. Today, a great effort is made to develop advanced *in silico* methods for predicting the metabolic properties of new chemical entities [15]. Such tools may model and/or predict a variety of issues related to metabolism, such as the site of metabolism (position of reactive atoms), metabolite structure, and the potency to inhibit or induct CYPs, major enzymes responsible for xenobiotic biotransformation. A variety of computational and statistical approaches can be used, including expert systems [16], data mining approaches [17], quantitative structure-activity relationships (QSARs) [18], machine learning-based methods [19], pharmacophore-based algorithms [20], shape-focused techniques [21], molecular interaction fields (MIFs) [22], reactivity focused techniques [23], protein-ligand docking, molecular dynamics (MD) simulations, and combinations of the abovementioned methods [15]. These approaches enable the prediction of metabolic issues relevant at early stages of the drug development pipeline and can lead to minimizing the risk related to an inappropriate metabolic and/or pharmacokinetic profile, which is one of the main reasons of a drug withdrawal from the market [24]. Some open-source metabolic software is available *e.g.* SMARTCyp [25], admetSAR [26], MetaPrint2D [17] as well as commercial ones: MetabolExpert (CompuDrug International, Bal Harbor, FL, USA) or Meteor Nexus (Lhasa Limited, Leeds, United Kingdom). Nevertheless, *in vitro* experiments followed by qualitative and quantitative analysis (mostly by LC-MS or LC-MS/MS) involving microsomes and cryopreserved or fresh hepatocytes are still a gold standard in early preclinical metabolic studies.

Among the available model systems suitable for *in vitro* metabolism simulation, liver microsomes are most widely used for high throughput screening of newly synthesized drug candidates [27]. Their main advantage is ease of the preparation procedure and a possibility of long-term storage, thus costs are dramatically reduced in comparison with cell- and animal-based assays. Moreover, microsomes are a rich source of all the main metabolizing enzyme types [28], from different families (I phase – CYPs, FMOs; II phase – UGTs). Typically, quantitative results are obtained with the use of liquid chromatography – mass spectrometry determination of a parent compound. This technique is well-established, routine in use and specific for the detection of a particular drug candidate with the help of the opportunity for structural identification *via* spectral data.

Mass spectrometry is a powerful technique, widely applied in structural studies, including drug metabolite identification [29], [30]. The structural information is obtained through a detailed interpretation of the fragmentation spectra recorded by MS/MS instruments. Sometimes, however, such MS/MS spectra are not enough to fully identify the chemical structure. In this case, there is a need to support MS data with additional experiments, for example magnetic nuclear resonance (NMR). NMR is still required to characterize the position of oxidation in an aromatic ring, the site of aliphatic oxidation, and to strictly locate other functional groups if fragmentation pathways are unavailable or inconclusive [31]. During early metabolic studies it is often difficult to obtain the required amount of metabolite with the suitable purity.

In the last decades quantitative structure-retention relationship (QSRR) methodology has gained popularity as a supportive tool in modeling pharmacological activity [32], the identification of peptides [33], [34], and most recently also in metabolomics [35] and for differentiation between isobaric compounds [36].

In this article, the possibility of an application of QSRR in order to support the hypothesis of the geometric isomerization of a set of newly synthesized sulfonamides, which was observed during an early *in vitro* metabolic stability study, is presented. Also the fragmentation pathways of some biotransformation products are proposed, and in the case of postulated isomerization, a predictive QSRR model is built. The proposed strategy is helpful at the early stages of drug development, especially within metabolic studies.

## Materials and Methods

### Reagents

A set of fifteen studied compounds were synthesized as described earlier [8] and used without further purification. Pooled human liver microsomes, NADPH, DMSO, formic acid and buffer constituents were purchased from Sigma-Aldrich (St. Louis, MO, USA). 18 MΩ grade water (Direct-Q 3 UV-R system, Merck, Darmstadt, Germany) was used. Acetonitrile and methanol were of LC-MS grade and were purchased from Merck (Darmstadt, Germany).

### Human Liver Microsomes (HLM) – *in vitro* model

Stock solutions of the studied compounds were made at a concentration of 1 mM in DMSO:MeOH mixture (10∶90 *v/v*) to prevent their precipitation. For biotransformation incubations, the solution of compounds was diluted to 10 µM with the presence of 1 mM of NADPH (Sigma-Aldrich, St. Louis, MO, USA) in a potassium phosphate buffer (0.1 M, pH 7.4). Incubation was carried out in thermoblock (Labnet International, Edison, NJ, USA) at 37°C and started with the addition of pooled human liver microsomes with a final concentration of 0.53 mg/mL (Sigma-Aldrich, St. Louis, MO, USA). Directly after HLM was added and at 120 min of incubation the reaction was ended by the addition of an equal volume of ice-cold methanol with 0.1% (*v/v*) of formic acid. The samples were immediately centrifuged (10 min at 11700 g) and the resulting supernatant was directly analyzed or kept at −80°C until LC-MS or LC-QTOF-MS analysis.

Additionally, chemical stability (negative control) in biotransformation experiment conditions was performed (phosphate buffer, 37°C, 2 h) to evaluate possible instability, unrelated to the activity of microsomes (including putative isomerization).

### Quantification of metabolic stability – LC-MS assay

LC-MS analysis was performed on an Agilent 1260 system coupled to a SingleQuad 6120 mass spectrometer (Agilent Technologies, Santa Clara, CA, USA). The amount of remaining parent compound was determined as a percentage of a peak area of a *trans* isomer at the start of the incubation.

A Poroshell C18 EC120 column (3.0×100 mm, 2.7 µm, Agilent Technologies, Santa Clara, CA, USA) was used in reversed-phase mode with gradient elution starting with 5% of phase A (0.1% formic acid in water) and 95% of phase B (0.1% formic acid in acetonitrile). The amount of phase B was linearly increased to 100% in 30 minutes. The total analysis time was 42 min at 25°C, the flow rate was 0.25 mL/min and the injection volume was 20 µL. The mass spectrometer was equipped with an electrospray ionization source and the ionization mode was positive. The mass analyzer was set individually to each derivative to detect pseudomolecular ions [M+H^+^]. The mass spectrometry detector parameters of the ESI source were as follows: nebulizer pressure 40 psi (N_2_), drying gas 10 mL/min (N_2_), drying gas temperature 300°C, capillary voltage +3 kV.

### Elucidation of metabolite structures – LC-MS/MS assay

The chromatographic method, providing the full separation of metabolites from a parent compound was transferred from the quantitative LC-MS assay. An Agilent 1100 chromatographic system (Agilent Technologies, Santa Clara, CA, USA) coupled to an ABSciex QSTAR XL (Applied Biosystems, Foster City, CA, USA) Q-TOF spectrometer was used to collect the fragmentation spectra of biotransformation products. An ESI source, operated in a positive ionization mode, was applied. The needle voltage was set at + 5.5 kV, the nebulizer gas at 20 psig, and the curtain gas flow rate at 10 L/min. The TOF analyzer was calibrated on a daily basis and subsequently operated at average measured accuracy of 0.05 Da. Data were collected at a rate of 1 spectrum per s. Preliminary MS/MS experiments were performed in the information-dependent acquisition (IDA) mode. One survey MS measurement was complemented with 2 data-dependent MS/MS measurements resulting in a cycle time of 5 s. Only singly charged precursor ions were selected, based on abundance. After being fragmented twice, a particular m/z value was excluded for 30 s. Selecting the same m/z value twice increases the chance of measuring a particular precursor at its maximum intensity while an exclusion time of 30 s allows MS/MS information to be obtained on chromatographically resolved isomers. The quadrupole was operated at unit resolution and the collision energy was set individually for the analytes. All data were acquired using Analyst QS 1.1 (Applied Biosystems, Foster City, CA, USA).

### QSRR – *in silico* model for isomer recognition

**Figure 2:**
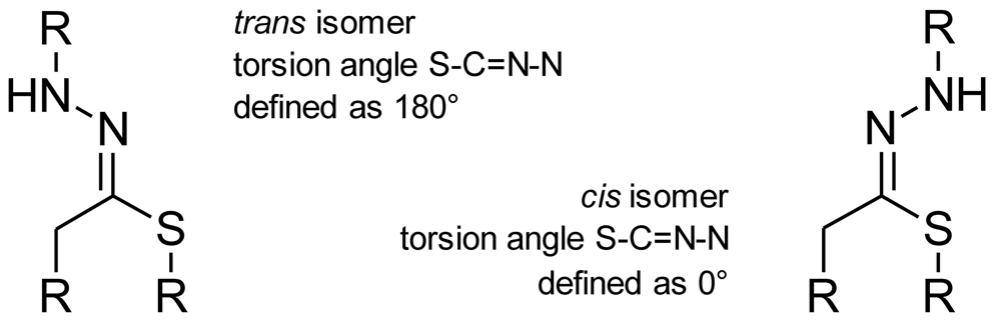
Definition of the torsion angle in *cis* and *trans* isomers of the studied benzensulfonamide derivatives.

QSRR methodology involved three main steps: molecular modeling (1a) along with descriptor calculations (1b), chromatographic experiments designed to assess lipophilicity parameters (2) and finally model building along with model validation (3).

(1a) The three-dimensional structure of the 15 studied compounds in *cis* as well as in *trans* forms (except compound 4, for which the putative *cis* peak was not observed) were built using the HyperChem (v. 8.0.3, HyperCube, Gainesville, FL, USA) graphical user interface, before starting molecular modeling algorithms in order to obtain the lowest energy geometry of a molecule. The dihedral (torsion) angle restraints were set up to enable a molecule to preserve its geometrical configuration during energy minimization calculations (Fig. 2). The two-dimensional model was first optimized with the molecular mechanics force field procedure (with the MM+ method, which has both a quadratic and a cubic stretch term in its potential, whereas other techniques, such as AMBER, OPLS, and BIO+ have only quadratic stretch terms). The resulting structures were further optimized by means of a semi-empirical method (with the use of the AM1 hamiltonian) and applying the Polak-Ribiere algorithm along with a gradient limit of 0.001 kcal•Å^−1^•mol^−1^. A similar approach was successfully applied in other reports [37].

(1b) Then, the minimum geometry structures were used to calculate molecular descriptors with the use of the Dragon software (Talete, Milano, Italy). Next, the software was used for initial dataset (29 compounds × 4885 descriptors) exploration with the help of the in-built PCA toolbox. Only those descriptor blocks that differentiate *cis-trans* isomers were used in further calculations: geometrical descriptors, 3D matrix-based descriptors, 3D autocorrelations, RDF descriptors, 2D-MoRSE descriptors, WHIM descriptors, GETAWAY descriptors, Randic molecular profiles, CATS 2D, 3D atom pairs. Regression calculations were performed with the help of Statistica (StatSoft, Tulsa, OK, USA).

(2) In order to obtain input lipophilicity data, reversed-phase liquid chromatography (RP-LC) log k_w_ determination experiments were performed using the Agilent 1260 apparatus (Agilent Technologies, Santa Clara, CA, USA) equipped with UV and single quadrupole mass detectors, and an autosampler and column thermostat. A Poroshell EC18 150×3 mm column with a particle size of 2.7 µm was applied. Chromatographic measurements were made at 30°C with an eluent flow rate of 0.25 mL/min. Two chromatographic runs were conducted for each compound with different linear gradient times (30 and 60 min), and with an acetonitrile composition in the mobile phase increasing from 5% to 100%. The obtained retention times were further used to calculate log k_w_ values applying the DryLab 2000 Plus software (LC Resources, Walnut Creek, CA, USA). DryLab estimated the log k_w_ value according to the linear equation: log k = logk_w_+Sφ (k – retention factor, S – slope, φ – volume fraction of the organic modifier) The elution program, as well as the column, mobile phase and instrument properties were taken into account to determine the coefficients in the abovementioned equation. Two analytical runs were required to fit the data, and peak assignment was done manually to ensure the correct identification. An assumption was made, that *trans* isomers are energetically more stable that *cis* isomers, hence they produce higher peaks on the chromatogram. This assumption does not apply to microsomal stability. The QSRR part involves studying compounds only in buffer solutions (without microsomes and NADPH), thus no biotransformation products were observed in these experiments.

(3) The QSRR model was built using the Statistica software (StatSoft, Tulsa, OK, USA). Multiple linear regression (MLR) was used. Log k_w_ was a dependent variable and the selected descriptors served as independent variables. The compounds were randomly divided into training (n = 24) and validation (n = 5) sets. Model coefficients were estimated using the training set only, while the rest of the compounds were used to show the ability to predict the log k_w_ value correctly. In order to avoid over-fitting a model, only 4 descriptors were finally incorporated into the model, using the forward stepwise method.

## Results and Discussion

The results are presented in two main subsections, involving the determination of metabolic stability, a proposal of possible biotransformation products (along with a tandem mass spectrometry spectra interpretation) and an evaluation of the usefulness of the QSRR tool.

### *In vitro* biotransformation of the studied compounds

**Figure 3:**
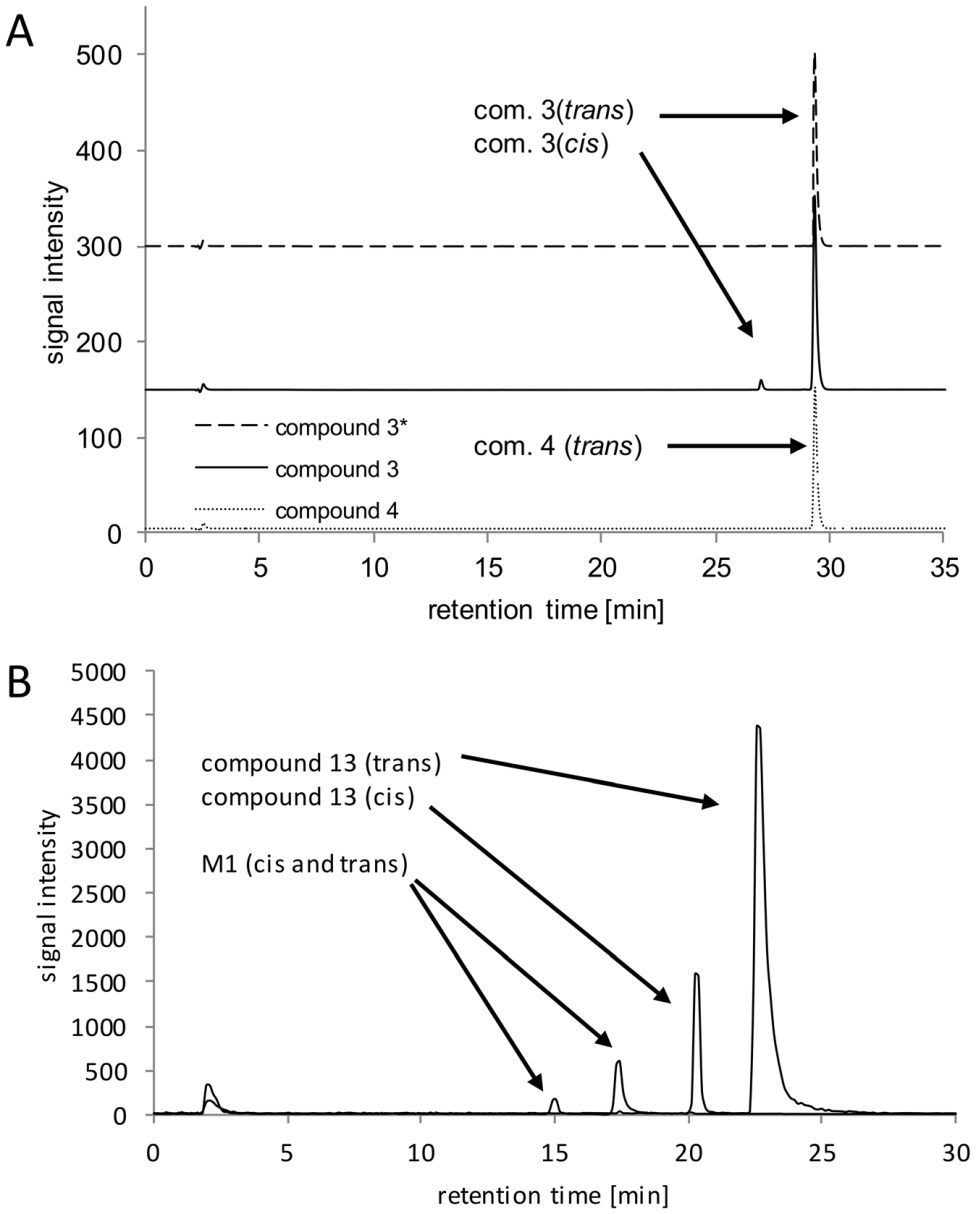
Exemplary liquid chromatography results: (A) Extracted ion chromatogram of water dissolved compounds 3 and 4; proposed peak assignment is presented, *compound 3 injected immediately after dissolution. (B) Extracted ion chromatograms of compound 13 and its oxidized biotransformation products after incubation in the presence of HLM and NADPH, proposed peak assignment is presented. Experimental details in the text.

The reversed-phase LC and LC-MS/MS chromatographic methods were successfully developed for the detection of 15 compounds from a new class of compounds demonstrating antiproliferative activity. The analytical method development stage included the appropriate stationary and mobile phase selection and an optimization of the gradient elution program in order to obtain the sufficient retention of all 15 compounds and a resolution suitable to resolve pairs of isomers and the formed biotransformation products. Exemplary chromatograms of compound 13 are presented in Fig. 3B.

An *in vitro* metabolic stability test utilizing rat liver microsomes along with a negative control (without NADPH and without NADPH and microsomes) revealed that most of the compounds are able to form an additional peak (Fig. 3A) in a chromatogram, giving ca 2–9% (relative abundance) of a new peak. Solutions in pure solvents analyzed by LC-MS immediately after the dissolution of the solid substance revealed high purity of the studied compounds. According to the chemical structure, *cis*-*trans* isomerization around the C = N bond in hydrazone moiety was proposed. Geometrical isomerism cannot be proved by mass spectrometry data. Surprisingly, isomerization in the studied conditions did not occur in the case of compound 4 (Fig. 3A), suggesting that an additional -NO_2_ group in a phenyl substituent strongly influences this reaction. This phenomenon is a subject of our further studies.

Additionally, the fragmentation pattern developed based on the recorded mass spectra enabled the structures of metabolites to be proposed.

Hydroxylation in the 5-chloro-4-methyl-2-sulfamoylphenyl ring (most probably in the methyl group) is a biotransformation pathway shared by all the studied compounds. For some compounds the relative abundance of the hydroxylated metabolite was low (Table 1) and did not exceed 3%. The proposed reason for such a situation is the presence of a phenyl ring (with more effect if it is substituted) directly to the hydrazone core. It should be noted that a comparison between compounds 8 and 9 revealed that the addition of a 2-carbon aliphatic linkage between hydrazone and phenyl results in a higher rate of biotransformation. Also all aliphatic substituted compounds were more susceptible to microsome catalytic activity.

**Table 1:** Summary of metabolic stability assay.

ID	% of compound remaining after 120 min incubation	% of isomerization at the beginning of incubation
**1**	53.51	5.84
**2**	75.13	6.52
**3**	76.13	7.76
**4**	62.86	0.00
**5**	79.95	5.84
**6**	83.88	3.50
**7**	60.90	5.53
**8**	58.23	5.25
**9**	96.03	9.28
**10**	90.80	7.79
**11**	98.97	1.50
**12**	95.11	5.41
**13**	95.33	2.66
**14**	74.88	0.00
**15**	99.40	4.93

**Figure 4:**
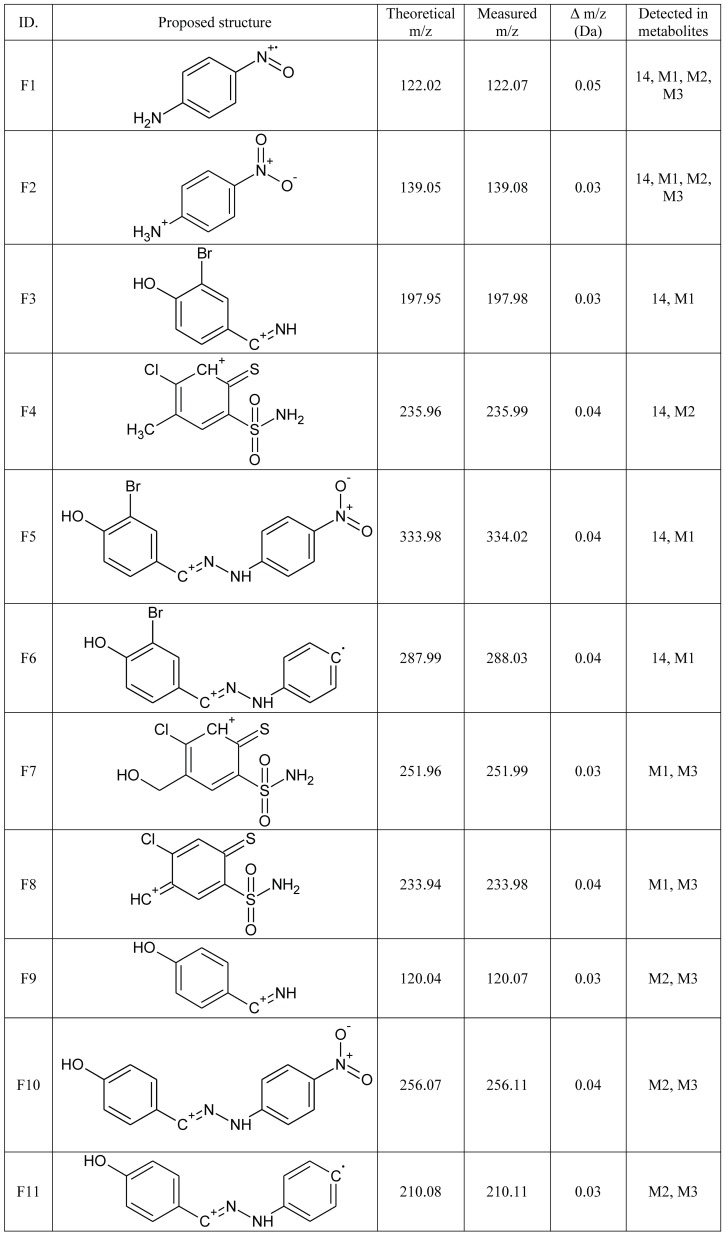
Common ions present in MS/MS fragmentation spectra of compound 14 and its three main biotransformation products. M1 – hydroxylated metabolite, M2 – debrominated metabolite, M3 – metabolite both hydroxylated and debromianted.

**Figure 5:**
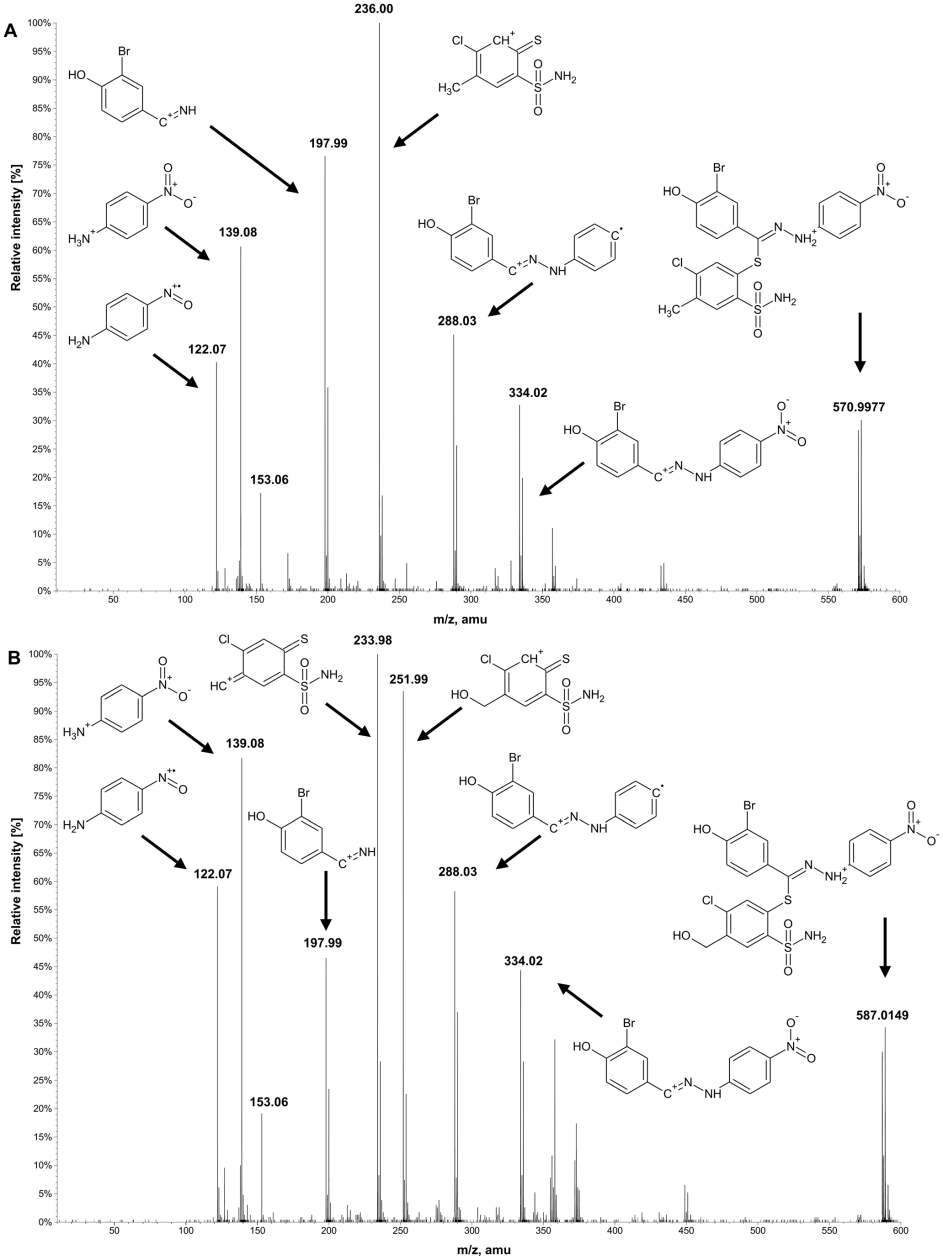
MS/MS fragmentation spectrum of protonated compound 14 (m/z 471, A) and metabolite M1 (m/z 587, B) along with proposed structures of formed fragment ions.

**Figure 6:**
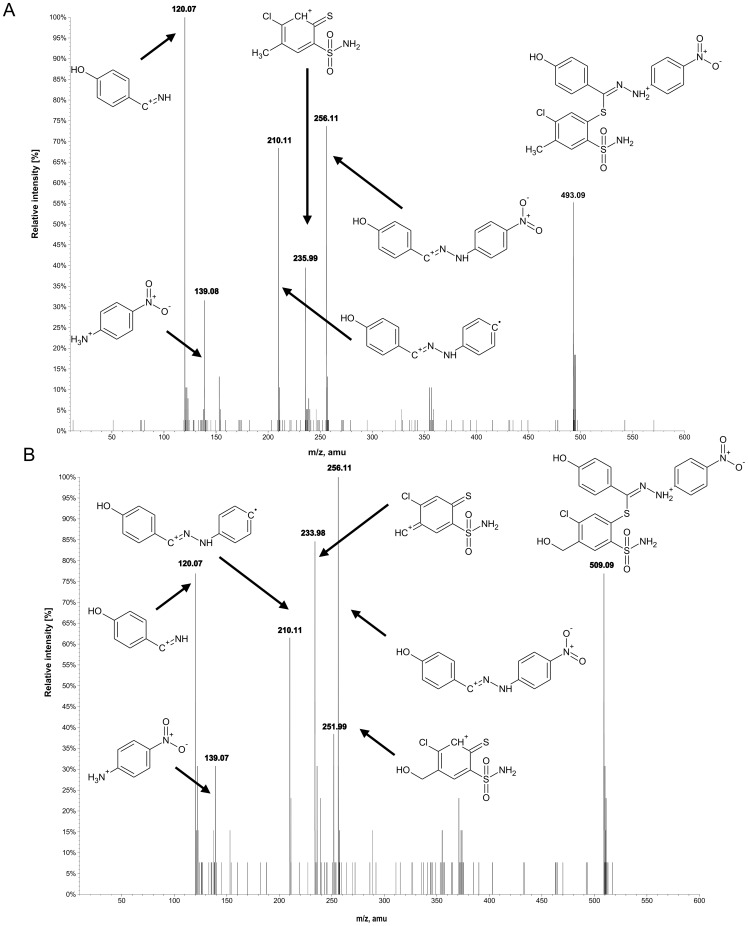
MS/MS fragmentation spectrum of protonated M2 (m/z 493, A) and M3 (m/z 509) metabolite of compound 14 along with proposed structures of formed fragment ions.

Most of the studied compounds are characterized by a good chemical and metabolic stability, even if the maximum incubation time (2 hours for microsomal preparations) was applied. For compound 14, a hydroxylation pathway (common for all derivatives) and a quite rare reductive debromination reaction, were observed. Below, a detailed fragmentation study of the products of its biotransformation is presented. Figure 4 gathers proposed structures of common ions along with m/z characteristics, whereas figures 5 and 6 present experimental spectra for compound 14 and its three main metabolites.

### Biotransformation of compound 14

**Figure 7:**
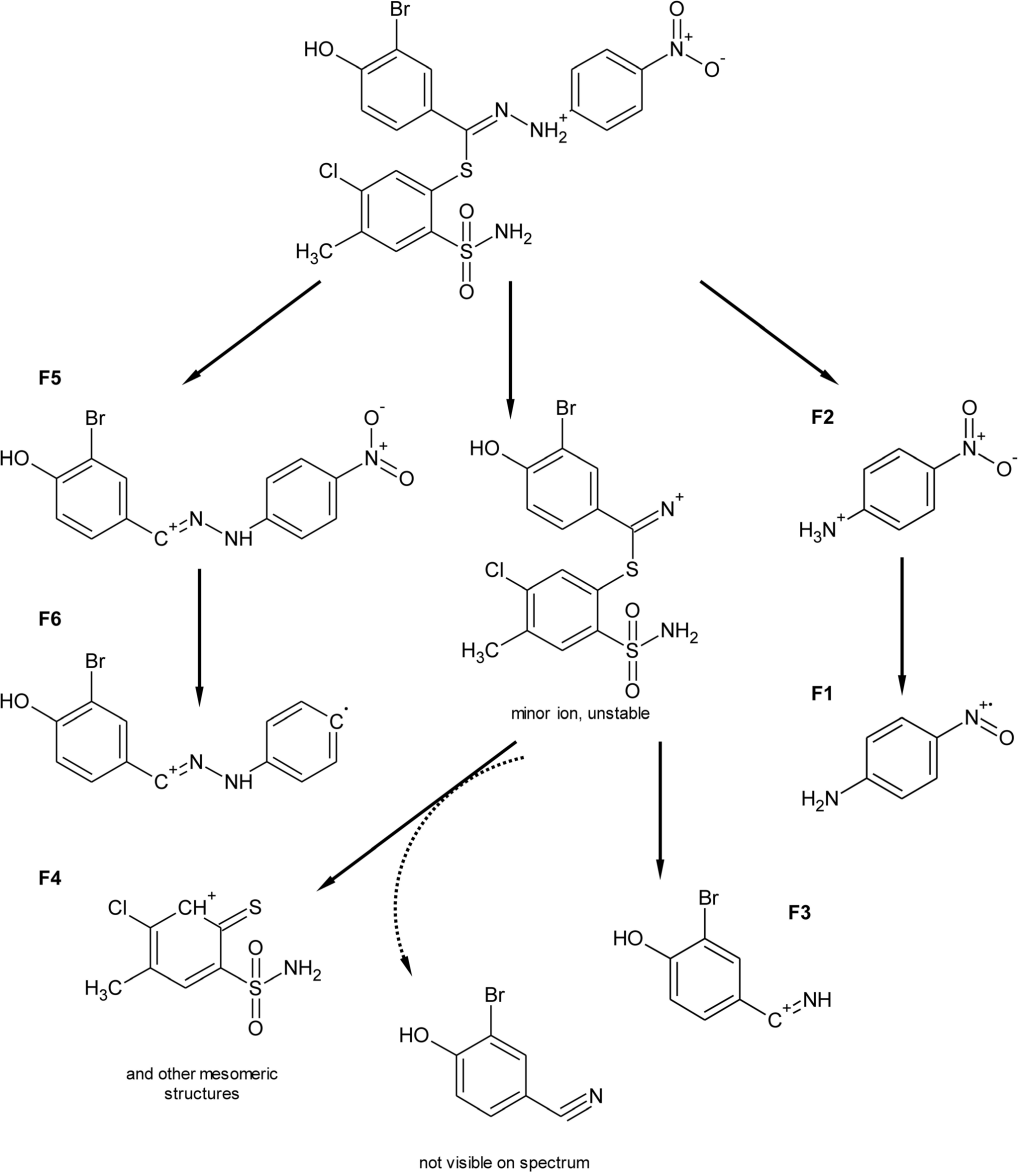
Proposed MS/MS fragmentation pathway of compound 14.

The identification and structural elucidation of compound 14 metabolites was performed based on the presence of product ions, in relation to the fragmentation of the parent compound. The fragmentation pattern was investigated by the fragmentation of the parent compound in a collision cell in the product ion mode of the mass spectrometer. The protonated molecule generates several product ions. The most abundant ions are F4 and F3 (Fig. 4), with an accurate mass of m/z 235.9950 and 197.9872, respectively. The fragmentation pattern is presented in Fig. 5. Ions F4 and F5 are formed by the breakage of the S-C bond in a tiohydrazone moiety, however, the formation of F4 is not a typical cleavage in fragmentation pathways. The formation of an unstable ion of m/z 434.9484 (poor signal abundance, Fig. 5A and Fig. 7) is probably followed by a release of stable 3-bromo-4-hydroxybenzonitrile (not visible in the spectrum) and the simultaneous formation of F4. Ions F1 and F3 are formed by the cleavage of the N-N bond. Ion F6 is formed from F5 after the loss of the NO_2_ radical [37]. The release of the OH radical can be also proposed for the formation of F1 from F2. Typically, the collision induced dissociation of pseudomolecular ions leads to the formation of even electron fragments, however, the odd electron species can also be formed [37]. The metabolites of other studied compounds present similar fragmentation pathways.

#### Hydroxylation (M1)

The oxidation of carbon atoms is one of the most common reactions catalyzed by CYP [38]. Monohydroxylated metabolite (M1) was found in a chromatogram at m/z 587.0149, [M+H]^+^. The detected product ions: F1, F2, F3, F5 and F6 (Fig. 4, 5B) suggest that hydrozone moiety is unchanged. It can be assumed that the hydroxylation is located on a benzensulfonamide ring, most probably in the methyl group, according to the most typical reaction catalyzed by CYP enzymes [38] and the presence of F7 and F8 ions on mass spectra. F8 is formed by the loss of water from ion F7, which is typical for hydroxylated C-OH bonds. Other, less probable structures include the direct hydroxylation of the phenyl ring or the S-oxidation of thiohydrazone moiety. The lack of the F4 ion in the fragmentation spectra of all +16 Da metabolites strongly supports the proposed -CH_3_ hydroxylation. However, the MS/MS fragmentation pattern did not allow full confirmation of this hypothesis. Several of the studied compounds were able to form two hydroxylated metabolites. The same overall mass, isotopic pattern and fragmentation pathways of both monohydroxylated metabolites suggest that they could be a pair of geometrical isomers. An exemplary chromatogram is shown for compound 13 (Fig. 3B).

#### Reductive debromination (M2)

Reductive dehalogenation, especially debromination, is a rarely reported metabolic reaction [39]. Reductive debromination is probably best documented for polybrominated diphenyl ethers (PBDEs), commonly used as flame retardants in the furniture, textile and electronics industries. Their metabolism was extensively studied using animal [40], [41] and plant [42] models, presenting subsequent debromination on a benzene ring. Here, a similar reaction is proposed, based on mass spectrometric data (Fig. 6A). A debrominated metabolite (M2) of a compound 14 peak was detected with an accurate m/z 493.0886 amu for the [M+H]^+^ ion. Product ions formed by the fragmentation of ion M2 support the hypothesis about the debromination of a molecule. The m/z values of 197.9872 (F3-Br), 288.0261 (F6-Br) and 334.0224 (F5-Br) especially provide strong evidence of debromination. One should also note that none of those ions are present in the mass spectrum of the parent compound, allowing the biotransformation origin of bromine loss to be confirmed.

#### Reductive debromination along with hydroxylation (M3)

A peak that corresponds to the hydroxylated and debrominated metabolite (M3, Fig. 6B) of compound 14 was also detected with an accurate m/z value of 509.0889 for the [M+H]^+^ ion. The fragmentation mass spectrum shares features characteristic for M1 (e.g. F8) and for M2 (e.g. F9, F10, F11). These results provide evidence of the subsequent occurrence of hydroxylation and debromination.

### Quantitative structure-retention relationships

Liquid chromatography is a technique used not only for quantitative and qualitative analysis but it can also serve as a tool for determining the dissociation constant and/or lipophilicity of analytes [43]. More recently the relevance of LC-based approaches has been emerging rapidly and QSRR models can support the identification of cellular metabolites in metabolomics of the protozoan parasite *Trypanosoma brucei* [35], drug structural isomers in human urine [36] or peptides in advanced proteomics studies [44].

The QSRR model is typically built on the base of calculated *in sillico*, so-called molecular descriptors, which are further preselected via a wide range of chemometric and statistical techniques in order to establish a regression equation, which should be characterized by good statistical properties and predictive power. The crucial issue is to mathematically describe the three-dimensional structure of geometrical isomers in such a way as to differentiate them, because most typical molecular descriptors used for retention prediction (*e.g.* clog P) have exactly the same value for a pair of *cis* and *trans* isomers. This limitation can be removed by the application of molecular descriptors derived from three-dimensional models of compounds. Dragon is an application frequently used to calculate molecular descriptors. Dragon descriptors can be used to evaluate molecular structure-activity (QSAR) or structure-property relationships (QSPR), chromatographic lipophilicity (log k_w_), among others. Dragon enables the calculation of almost 5000 molecular descriptors, which are divided into 29 logical blocks, each in turn divided into a number of sub-blocks. The software requires a chemical structure file as an input. The HyperChem output – hin file, is a three-dimensional representation of a molecule, which contains geometry coordinates as well as partial charges of all atoms. An extensive review of all the molecular descriptors, their mathematical definitions and properties are reported in an excellent book [45], which contains also a detailed reference list.

Purity of the studied compounds, understood as the presence of some by-products or degradation products was proven earlier by NMR analysis in a DMSO solution [8]. Any impurities that contain different atom compositions were excluded also by MS/MS data. Since no additional peaks are observed for freshly prepared solutions, it was postulated that the studied compounds were pure in solid form and were subjected to the isomerization process after dissolution, unrevealed in the mechanism.

Double peaks corresponding to all compounds (except compound 4) were detected; large peaks are proposed to be *trans*-isomers and small peaks just before the *trans*-isomers are proposed to be *cis*-isomers. While only one peak corresponding to the *trans*-isomer appears for immediately analyzed solutions of compounds in a pure solvent, additional small peaks of *cis*-isomers are detected in all biotransformation samples, which require some necessary sample preparation steps – and thus – time. Since additional peaks are present in all solutions of drugs, it is believed that the studied thiohydrazones isomerize quickly when dissolved. None of the checked solvents compatible with liquid chromatography – mass spectrometry (methanol, acetonitrile, deionized water) were able to stop isomerization

**Figure 8:**
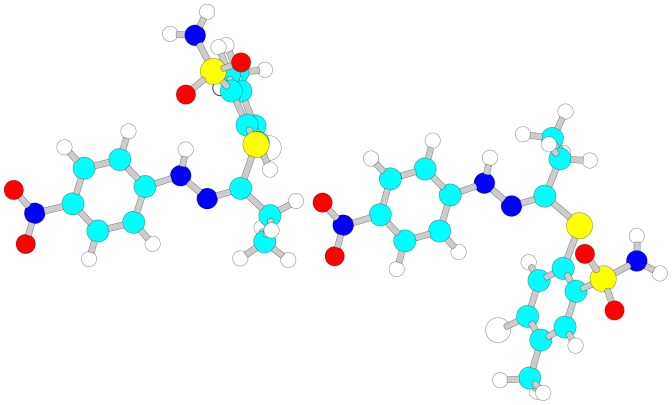
3-dimentional structures of compound 1 isomers obtained after optimization in the HyperChem software.

The QSRR model was built on the basis of log k_w_ (modeled property) values determined on a Poroshell EC18 column using a method implemented in the DryLab software. As it is presented in Table 2, the log k_w_ values are different for *cis* and *trans* isomers. This means that the chromatographic system in this case can be considered as a probe, which recognizes not only the polarity of a compound but also its geometrical shape. The *cis* isomer for compound 4 was not observed (Fig. 3A), hence this isomer was not included in the calculations. An exemplary pair of three-dimensional structure models for compound 1 is shown in Fig. 8.

**Table 2:** Summary of retention data obtained for 15 studied compounds and their isomers.

Compound	Retention time30 min gradient [min]	Retention time60 min gradient [min]	S-value	logk_w_
**1-*trans***	27.97	45.94	4.64	3.60
**1-*cis***	26.03	42.34	5.00	3.52
**2-*trans***	29.45	48.28	4.04	3.39
**2-*cis***	27.54	44.88	4.43	3.40
**3-*trans***	29.35	48.37	4.30	3.56
**3-*cis***	27.00	43.98	4.62	3.44
**4-*trans***	29.37	48.51	4.41	3.64
**5-*trans***	30.36	50.32	4.22	3.64
**5-*cis***	28.50	46.83	4.47	3.56
**6-*trans***	30.52	50.56	4.16	3.62
**6-*cis***	28.06	45.89	4.40	3.45
**7-*trans***	31.83	53.19	4.17	3.80
**7-*cis***	29.97	49.67	4.37	3.69
**8-*trans***	30.32	50.30	4.28	3.68
**8-*cis***	29.10	47.99	4.41	3.60
**9-*trans***	29.80	49.14	4.18	3.54
**9-*cis***	27.75	45.36	4.49	3.46
**10-*trans***	31.60	52.64	4.08	3.70
**10-*cis***	28.95	47.73	4.46	3.62
**11-*trans***	29.18	48.14	4.41	3.61
**11-*cis***	27.52	45.08	4.70	3.57
**12-*trans***	31.11	51.63	4.05	3.62
**12-*cis***	28.80	47.32	4.35	3.52
**13-*trans***	29.41	48.44	4.25	3.54
**13-*cis***	27.91	45.69	4.51	3.50
**14-*trans***	26.77	43.63	4.76	3.49
**14-*cis***	25.34	40.95	4.99	3.41
**15-*trans***	32.03	53.49	4.07	3.75
**15-*cis***	29.67	49.09	4.37	3.66

Table 2 gathers the values of log k_w_, and obviously shows that all the studied compounds except for compound 4 (Fig. 3A) exist in a water solution as a pair of isomers, and their retention factors differ significantly. Typically the retention time is reported as a derivative of the lipophilicity, shape and electronic properties of a compound. Molecular descriptors are usually used to describe those properties. In order to calculate descriptors one needs to have three-dimensional models of compounds. The Dragon software enables over 4 thousand diverse descriptors to be calculated. Among them, those which are able to differentiate *cis* and *trans* isomers were carefully selected based on preliminary principal component analysis (data not shown). 1121 descriptors from the groups: geometrical descriptors, 3D matrix-based descriptors, 3D autocorrelations, RDF descriptors, 2D-MoRSE descriptors, WHIM descriptors, GETAWAY descriptors, Randic molecular profiles, CATS 2D and 3D atom pairs were selected and used in a stepwise regression analysis. For good statistical significance, the calculations were stopped when the four best descriptors gave the equation:
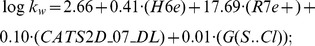
n = 24, R^2^ = 0.90, F = 52.43, p<1×10^−4^, s = 0.036,

H6e – H autocorrelation of lag 6 / weighted by Sanderson electronegativity (GETAWAY descriptors), p = 1×10^−9^;

R7e+ – R maximal autocorrelation of lag 7 / weighted by Sanderson electronegativity (GETAWAY descriptors), p = 2.1×10^−5^;

CATS2D_07_DL – CATS2D Donor-Lipophilic at lag 07 (CATS 2D descriptors), p = 4×10^−9^;

G(S..Cl) – sum of geometrical distances between S..Cl, (3D atom pairs), p = 4.5×10^−4^.

**Figure 9:**
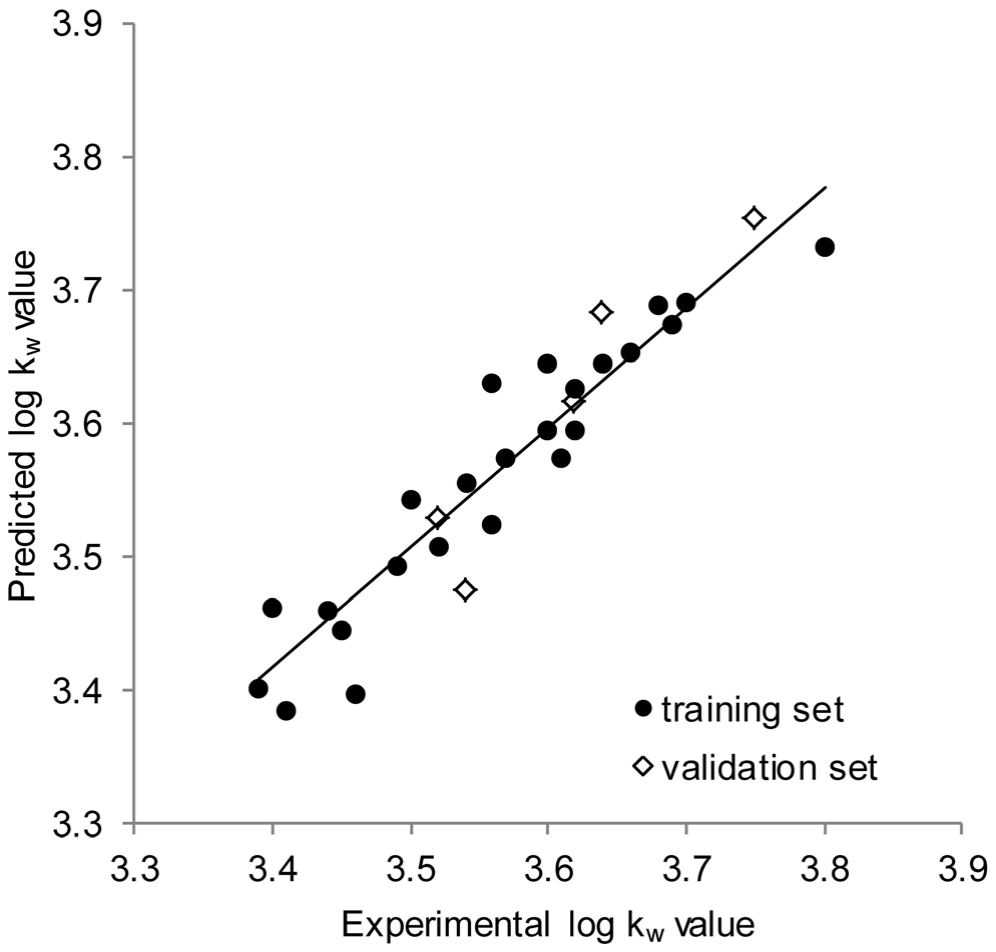
Correlation between experimental log k_w_ values and those predicted by the developed model.

As can be seen from the statistical properties of the obtained equation, the model is statistically significant, it explains a 90% variation of the dataset, and is characterized by a very small standard error of estimation (s), namely 0.036, which can be observed also on the correlation chart in Fig. 9.

GETAWAY descriptors (H6E, R7e+, in this model) were introduced in 2002 by Consonni and co-workers [46]. They are mathematical quantities calculated from a molecular representation depending on the geometry, the so-called molecular influence matrix and the influence/distance matrix. Calculated values can be weighted by the diverse molecular properties of atoms, *e.g.* atomic mass, polarizability, van der Waals volume, and electronegativity. In this case, two descriptors weighted by electronegativity have been included in the model. Probably electronegativity influences both the polarity of a molecule and the dependence between the shape and the dipole moment, thus has a consequence in the chromatographic interaction and altered retention of isomer pairs. The impact of these two descriptors is crucial to differentiate isomer pairs.

Moreover, also the distance between the H-donors and lipohilic parts of a molecule influences the retention of the studied compounds. Surprisingly, the G(S..Cl) descriptor, which is calculated as a sum of geometrical distances between S and Cl, suggests that the benzensulfonamide ring along with chlorine and the sulfur atom from tiohydrazone moiety is responsible for some crucial interactions influencing retention. It should also be noticed that compound 10 (the additional Cl atom in R1 substituent) is characterized by the biggest difference in retention time of the isomer pair; those two compounds fit the model with a very small error of estimation.

Geometrical isomerism within organic compounds and biomolecules was extensively reviewed in an article by Dugave and Demange [47]. Hydrazones display properties similar to those of imines, in particular *cis*-*trans* isomerism. The presence of an additional nitrogen atom (C = N-N) decreases the double-bond character of the π-system (C = N) and facilitates isomerization. Hydrazone can undergo both photo and thermal isomerization, which is usually facilitated in polar solvents, by acid/base catalysis and by electron-donating substituents. In most cases, the lowest energy configuration is E or *trans* [47]

Terhorst and Jorgensen studied conformational equilibria of 18 prototypical organic molecules, hydrazones, among others [48]. Quantum mechanical calculations were made. A rational explanation of the differences in free energy based on the steric and electronic effects was proposed. For hydrazones (N-methyl hydrazone of acetaldehyde and acetone) *trans* isomers were found to be a lower energy species, with the obvious influence of steric interactions. Molecular modeling calculations for novel thiohydrazones studied in this paper revealed similar results. The total energy difference for *cis* and *trans* isomers of compound 3 was found to be 2.65 kcal/ml, suggesting that the *trans* isomer is more preferable energetically.

Moreover, a similar occurrence of some additional peaks on chromatograms for hydrazone compounds was noticed earlier [49]. In the cited study, a proposal of salicylaldehyde isonicotinoyl hydrazone derivatives isomerism occurring in a water solution was made. A high purity of the compounds was proven by NMR and by a comparison of the retention times of the putative precursor, by-products and degradants. Finally, the MS/MS experiment revealed that newly formed peaks produce the same precursor ions and the same fragment ions as the parent compounds. Any attempts to apply preparative chromatography to purify a substance under an additional peak failed, because the *cis* isomer is less stable and converts in the solid state into the *trans*-isomer.

The abovementioned facts suggest that molecular modeling along with chemometric QSRR analysis led to an equation that strongly supports the hypothesis of the geometric isomerization of the fourteen studied compounds. The small error of estimation and the unsupervised selection of four molecular descriptors with quite an easy to explain physical explanation in the view of modern chromatography theory, allows the identification of additional peaks on the chromatogram to be considered as most probable.

## Conclusions

In this study, the supportive role of the QSRR model during structure elucidation within the biotransformation products of a series of potentially anticancer sulfonamide derivatives, in the case when tandem mass spectrometry fails to distinguish between isomers, and in the case when there is lack of synthetic standards of both isomers, is presented. Microsome incubations were carried out in the presence of NADPH as a cofactor and followed by LC-MS. From the biochemical point of view it is concluded that novel thiohydrazone moiety is stable under the studied conditions, and the considered compounds are susceptible to hydroxylation, most probably in the methyl group. Compound 14 undergoes a reductive debromination reaction, which is a rather rare biotransformation, cited only a few times in literature. On the other hand, additional peaks on the chromatogram, characterized by the same spectral properties as parent compounds, are hypothesized to be products of isomerization without any influence of the *in vitro* metabolism model. The usefulness of the proposed approach based on molecular modeling and the QSRR methodology, which can support the hypothesis of geometric isomerization, is also shown. The obtained model is statistically significant, successfully validated by an external set of compounds, and has a reasonable physico-chemical explanation. Although a definite assignment of peaks and molecule geometry was not provided by other recognized method, our results indicate that the model of chromatographic retention can successfully support structural identification in cases when the most frequently used mass spectrometry techniques are not informative enough.
